# The SARS-CoV-2 Delta-Omicron Recombinant Lineage (XD) Exhibits Immune-Escape Properties Similar to the Omicron (BA.1) Variant

**DOI:** 10.3390/ijms232214057

**Published:** 2022-11-14

**Authors:** Prerna Arora, Lu Zhang, Cheila Rocha, Luise Graichen, Inga Nehlmeier, Amy Kempf, Anne Cossmann, Gema Morillas Ramos, Eva Baier, Björn Tampe, Onnen Moerer, Steffen Dickel, Martin S. Winkler, Georg M. N. Behrens, Stefan Pöhlmann, Markus Hoffmann

**Affiliations:** 1 Infection Biology Unit, German Primate Center—Leibniz Institute for Primate Research, Kellnerweg 4, 37077 Göttingen, Germany; 2 Faculty of Biology and Psychology, Georg-August-University Göttingen, Wilhelmsplatz 1, 37073 Göttingen, Germany; 3 Department for Rheumatology and Immunology, Hannover Medical School, Carl-Neuberg-Straße 1, 30625 Hannover, Germany; 4 German Centre for Infection Research (DZIF), Partner Site Hannover-Braunschweig, Carl-Neuberg-Straße 1, 30625 Hannover, Germany; 5 Department of Nephrology and Rheumatology, University Medical Center Göttingen, Georg-August University of Göttingen, Robert-Koch-Straße 40, 37075 Göttingen, Germany; 6 Department of Anesthesiology, University Medical Center Göttingen, Georg-August University of Göttingen, Robert-Koch-Straße 40, 37075 Göttingen, Germany; 7 Centre for Individualised Infection Medicine (CiiM), Feodor-Lynen-Straße 7, 30625 Hannover, Germany

**Keywords:** SARS-CoV-2, delta variant, omicron variant, delta-omicron, deltacron, recombinant, spike protein, BA.1, antibody evasion, neutralization

## Abstract

Recently, a recombinant SARS-CoV-2 lineage, XD, emerged that harbors a spike gene that is largely derived from the Omicron variant BA.1 in the genetic background of the Delta variant. This finding raised concerns that the recombinant virus might exhibit altered biological properties as compared to the parental viruses and might pose an elevated threat to human health. Here, using pseudotyped particles, we show that ACE2 binding and cell tropism of XD mimics that of BA.1. Further, XD and BA.1 displayed comparable sensitivity to neutralization by antibodies induced upon vaccination with BNT162b2/Comirnaty (BNT) or BNT vaccination followed by breakthrough infection. Our findings reveal important biological commonalities between XD and Omicron BA.1 host cell entry and its inhibition by antibodies.

## 1. Introduction

A hallmark of the COVID-19 (coronavirus disease 2019) pandemic is the constant emergence of new SARS-CoV-2 lineages, some of which have increased transmissibility and/or reduced sensitivity to antibody-mediated neutralization. The first months of the pandemic were dominated by early descendants of the SARS-CoV-2 B lineage, including B.1, which did not greatly differ from the first SARS-CoV-2 isolates (such as Wuhan-Hu-01) on the genomic level. However, in late 2020, three SARS-CoV-2 lineages emerged, which evolved mutations that led to significantly elevated transmissibility (B.1.1.7, Alpha variant) and/or increased neutralization resistance (B.1.351, Beta variant and P.1, Gamma variant) and thus were termed variants of concern (VOC) [1,2,3].

A fourth VOC, the SARS-CoV-2 Delta variant (B.1.617.2 and sublineages), was first detected in India causing a massive surge in cases between March and May 2021, and subsequently became dominant worldwide [4,5]. Compared to previous SARS-CoV-2 variants, the Delta variant had increased replicative fitness [4,6] and patients infected by the Delta variant were more likely to require hospitalization [7]. The dominance of the Delta variant lasted until late 2021 when the highly mutated Omicron variant (B.1.1.529 and sublineages) emerged in South Africa [8]. Initially, three Omicron sublineages, BA.1, BA.1.1, and BA.2, co-circulated with and finally outcompeted the Delta variant globally. More recently, novel Omicron sublineages (including BA.2.12.1, BA.2.75, BA.4, BA.4.6, and BA.5) emerged and became dominant in multiple countries. All Omicron sublineages share a markedly reduced sensitivity to neutralization by antibodies elicited after infection by previous variants or vaccination [4,5,9,10,11,12,13]. Importantly, when compared to the Delta variant, the Omicron variant appears to cause less severe disease in most infected individuals, which may stem from the lower intrinsic virulence of the Omicron variant and the fact that a higher proportion of people have preexisting immunity from previous infection or vaccination [14,15,16].

**Figure 1:**
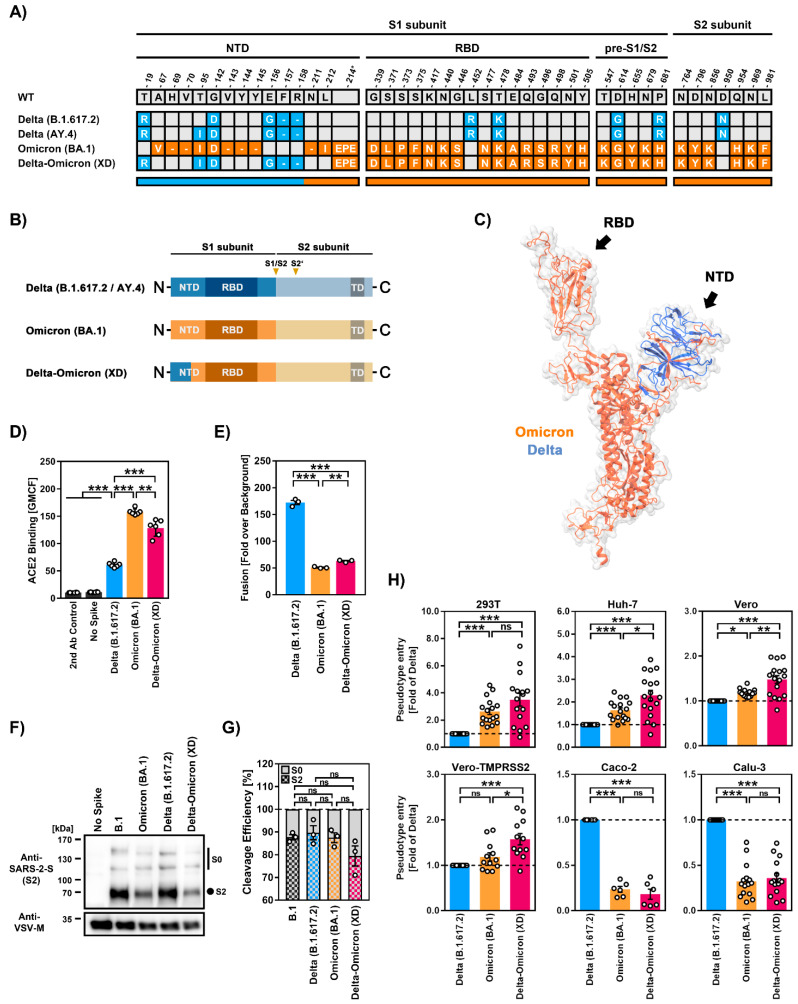
Host cell tropism, ACE2 binding, cell–cell fusion, and cleavage efficiency by SARS-CoV-2 Delta-Omicron (XD) recombinant lineage. (**A**) Summary of mutations present in the different SARS-CoV-2 S proteins (numbering according to the S protein of SARS-CoV-2 Wuhan-Hu-01). S protein mutations found in variants B.1.617.2, AY.4 (both Delta, blue), BA.1 (Omicron, orange), and XD (Delta-Omicron, blue and orange) are highlighted. Abbreviations: NTD = N-terminal domain; RBD = receptor-binding domain. (**B**) S protein domain structure. Abbreviations: TD = transmembrane domain; S1/S2 and S2′ = cleavage sites in the S protein. (**C**) Three-dimensional structure of monomeric XD S protein in which the regions originating from either the Delta (blue) or Omicron (orange) S protein are highlighted. (**D**) Soluble ACE2 binding by the indicated S proteins was analyzed by flow cytometry. Cells incubated with secondary antibody alone served as controls. Data represent mean ± SD geometric mean channel fluorescence from six biological replicates. Statistical analysis was performed using two-tailed Student’s *t*-test (**, *p* ≤ 0.01; ***, *p* ≤ 0.001). (**E**) S protein-driven cell–cell fusion was analyzed using a beta-galactosidase reconstitution assay. Data represent mean ± SEM cell–cell fusion (normalized against the assay background, no S protein) from three biological replicates, each carried out with four technical replicates. Statistical analysis was performed using two-tailed Student’s *t*-test (**, *p* ≤ 0.01; ***, *p* ≤ 0.001). (**F**) S protein particle incorporation and cleavage efficiency were analyzed by immunoblot. Data represent a single biological replicate and results were confirmed in two additional experiments. (**G**) Quantification of S protein cleavage. Data represent mean ± SEM cleavage efficiency (normalized against total S protein) from three biological replicates. Statistical analysis was performed using two-tailed Student’s *t*-test (not significant [ns], *p* > 0.05). Abbreviations: S0, uncleaved S protein; S2, S2 subunit of cleaved S protein. (**H**) S protein-driven cell entry was analyzed by transduction of the indicated target cell lines using pseudotype particles bearing the respective S proteins. Data represent mean ± SEM cell entry efficiency (normalized for Delta) from six to twelve biological replicates (each with four technical replicates). Statistical analysis was performed using two-tailed Student’s *t*-test (ns, *p* > 0.05; *, *p* ≤ 0.05; **, *p* ≤ 0.01; ***, *p* ≤ 0.001). Please also see Figure S2 for representative unprocessed pseudotype entry data.

Recently, infections caused by a recombinant SARS-CoV-2 lineage, which contains genomic regions of the Delta and Omicron variant, were documented in France [17,18] and other countries worldwide (Figure S1). This virus was tentatively named “Delta-Omicron” or “Deltacron” and was assigned the PANGO lineage XD. The main portion of the XD genome originates from the Delta variant (sublineage AY.4). However, most of the spike (S) protein open reading frame has been exchanged by the corresponding sequence of the Omicron variant (sublineage BA.1) [18,19], including the receptor-binding domain, a key target of antibodies (Figure 1A–C). The SARS-CoV-2 S protein is responsible for host cell entry and the main target of neutralizing antibodies. In the case of the Omicron variant, the S protein harbors more than 30 mutations, which modulate cell tropism and likely transmissibility and markedly reduce sensitivity to antibody-mediated neutralization [10,11,12,13]. Consequently, the emergence of the XD lineage raised concerns, as such a recombinant virus may unite the elevated pathogenicity of the Delta variant with the high transmissibility and antibody evasion properties of the Omicron variant. However, entry factor usage and cell tropism of the XD lineage have not been analyzed, and limited data are available on the blockade of this process by neutralizing antibodies [20,21].

Here, we compared the S proteins of the recombinant SARS-CoV-2 XD lineage and the Delta (B.1.617.2) and Omicron (BA.1) variants for their ability to bind ACE2, mediate virus–cell and cell–cell fusion, and their sensitivity to antibody-mediated neutralization. For this, we employed pseudovirus particles bearing the respective S protein, which represent an adequate surrogate system for studying SARS-CoV-2 host cell entry and its neutralization [22].

## 2. Results

### 2.1. The S Protein of the Recombinant SARS-CoV-2 Lineage XD Efficiently Binds ACE2 but Has Low Capacity to Drive Cell–Cell Fusion

We first investigated the XD S protein for its ability to bind ACE2 using a soluble human ACE2-Fc construct and flow cytometry. Compared to Delta (B.1.617.2)-S, both Omicron (BA.1)-S and Delta-Omicron (XD)-S bound ACE2 with higher efficiency, although ACE2 binding of Delta-Omicron (XD)-S was slightly less efficient as compared to Omicron (BA.1)-S (~1.2-fold reduction) (Figure 1D). Next, we studied S protein-driven cell–cell fusion, which is believed to contribute to COVID-19 pathogenesis [23,24]. The expression of Delta (B.1.617.2)-S induced strong cell–cell fusion, which was significantly higher compared to Omicron (BA.1)-S (Figure 1E), as expected [4,25]. Cell–cell fusion driven by Delta-Omicron (XD)-S was also reduced compared to Delta (B.1.617.2)-S but was slightly higher as compared to Omicron (BA.1)-S (Figure 1E). 

### 2.2. Similar Cell Line Tropism by SARS-CoV-2 XD and BA.1 (Omicron Variant) Lineages

Cleavage of the SARS-CoV-2 S protein at the border between the S1 and S2 subunits by the host cell protease furin is required for efficient entry into lung cells [26]. Therefore, we analyzed the cleavage efficiency of pseudovirus-incorporated S proteins by immunoblot but did not find significant differences between the tested S proteins, although a minor tendency for decreased cleavage of Delta-Omicron (XD)-S compared to Delta (B.1.617.2)-S and Omicron (BA.1)-S was noted (Figure 1F,G). With respect to S protein-driven entry into cell lines routinely used for SARS-CoV-2 research, we observed a general correlation between entry efficiency of particles bearing Omicron (BA.1)-S (BA.1_pp_) or Delta-Omicron (XD)-S (XD_pp_). For instance, cell entry of BA.1_pp_ or XD_pp_ was higher compared to particles bearing Delta (B.1.617.2)-S (Delta_pp_) for 293T (human kidney), Huh-7 (human liver), and Vero cells (African green monkey kidney), while the opposite trend was observed for Caco-2 (human colon) and Calu-3 cells (human lung) (Figure 1H). Furthermore, we noted a subtle (but significant) increase in cell entry of XD_pp_ over cell entry of BA.1_pp_ for Huh-7, Vero, and Vero-TMPRSS2 cells (Vero cells that stably overexpress the S protein-activating protease TMPRSS2) (Figure 1H).

### 2.3. Diminished Sensitivity to Antibody-Mediated Neutralization by the Recombinant SARS-CoV-2 XD Lineage

Finally, we examined the sensitivity of particles bearing Delta-Omicron (XD)-S to neutralization by antibodies induced by vaccination with either two or three doses of the BNT162b2/Comirnaty vaccine and breakthrough infection in vaccinated individuals during the “Delta wave” (10/2021-01/2022) or “early Omicron wave” (02/2022-05/2022, dominated by BA.1 and BA.2) in Germany. 

**Figure 2:**
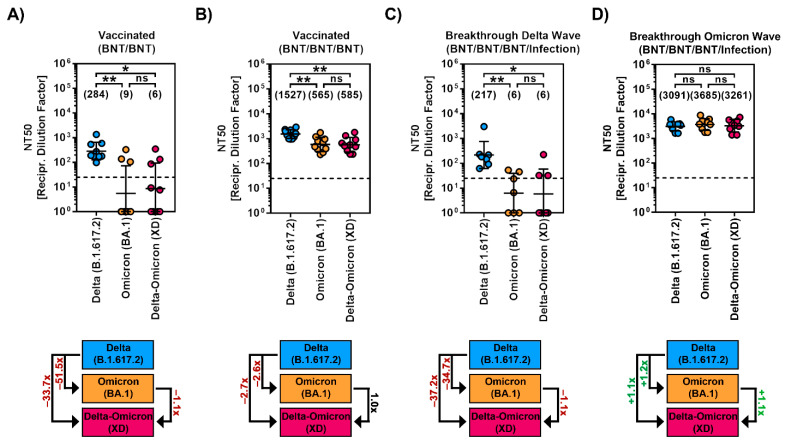
Immune evasion by SARS-CoV-2 Delta-Omicron (XD) recombinant lineage. (**A**) Particles bearing the indicated S proteins were incubated with plasma from individuals that received two doses of the Cormirnaty/BNT162b2 vaccine, before being added to Vero cells. Pseudotype entry was normalized against the respective control (set as 0% inhibition). Further, the neutralizing titer that reduced pseudotype entry by 50% (NT50) was calculated (of note, samples with NT50 < 6.25 were considered negative and values were manually set as 1). In addition, the median fold change in NT50 was calculated (for comparison of BA.1_pp_ and XD_pp_ neutralization, only samples with NT50 (BA.1_pp_) > 6.25 were selected). Presented are the geometric mean NT50 data (indicated by black lines and numerical values). (**B**) The experiment was performed as described in panel A but plasma from ten individuals that received three doses of the Cormirnaty/BNT162b2 vaccine was used. (**C**) The experiment was performed as described in panel A but serum from seven individuals that received three doses of the Comirnaty/BNT162b2 vaccine and that were infected during the “Delta wave” in Germany was used. (**D**) The experiment was performed as described in panel A but plasma from nine individuals that received three doses of the Comirnaty/BNT162b2 vaccine and that were infected during the “Omicron wave” in Germany was used. Statistical analysis was performed using Kruskal–Wallis analysis with Dunns’ multiple comparison test (ns, *p* > 0.05; *, *p* ≤ 0.05; **, *p* ≤ 0.01). Please also see Figure S3 for individual neutralization data.

For plasma collected after two doses of BNT162b2/Comirnaty (BNT/BNT), we observed strongly reduced neutralizing activity (~51.5-fold reduced) against BA.1_pp_ compared to Delta_pp_, which is in line with previous reports [11,12,13], and neutralizing activity against XD_pp_ was similar to that against particles bearing Omicron BA.1_pp_ (Figure 2A). Plasma obtained after three doses of the BNT162b2/Comirnaty vaccine (BNT/BNT/BNT) showed generally increased neutralizing activity and neutralized all particles efficiently, with neutralization being highest for Delta_pp_ and only slightly but comparably reduced for BA.1_pp_ or XD_pp_ (~2.6–2.7-fold reduction) (Figure 2B). Neutralizing activity in sera collected from BNT/BNT/BNT vaccinated individuals that were infected during the “Delta wave” was similar that of BNT/BNT/BNT vaccinated individuals without breakthrough infection, showing strongly reduced neutralizing activity against BA.1_pp_ (~34.7x reduced) and XD_pp_ (~37.2x reduced) compared to Delta_pp_ (Figure 2C). Finally, plasma collected from BNT/BNT/BNT-vaccinated individuals that were infected during the “early Omicron wave” neutralized Delta_pp_, BA.1_pp_ and XD_pp_ with comparable efficiency (Figure 2D).

## 3. Discussion

Here we show that the S proteins of the recombinant XD lineage of SARS-CoV-2 and the Omicron (BA.1) variant share important biological traits, i.e., comparable ACE2 binding, cell–cell fusion, cell line tropism, and sensitivity to antibody-mediated neutralization. 

Our results are in accordance with the S proteins of XD and BA.1 sharing high sequence identity, with only the first half of the N-terminal domain (NTD) originating from Delta (AY.4)-S and the remainder stemming from BA.1 S, as discussed below. However, we observed subtle differences between Omicron (BA.1)-S and Delta-Omicron (XD)-S with respect to ACE2 binding (slightly reduced for Delta-Omicron (XD)-S) and cell line tropism (slightly increased Huh-7, Vero, and Vero-TMPRSS2 cell entry for particles bearing Delta-Omicron (XD)-S), which may reflect minor changes in the ability of the Delta-Omicron (XD)-S to enter target cells, perhaps due to slight differences in the interactions with attachment factors such as neuropilin-1 or heparan sulfate [27,28]. 

We found no appreciable differences in the neutralization sensitivity of XD_pp_ and BA.1_pp_ to antibodies induced upon BNT162b2/Comirnaty (BNT) vaccination or BNT vaccination followed by breakthrough infection. This finding is in agreement with two recent studies [20,21] and reflects that a substantial portion of neutralizing antibodies target the RBD of the S protein, which is identical between BA.1 and XD S proteins. Neutralizing antibodies can also be directed against the NTD, which comprises 330 amino acids (based on the Wuhan-Hu-01 isolate). In the XD S protein, the first half of the NTD is derived from the Delta S protein while the second half of the NTD stems from the Omicron BA.1 S protein (of note, due to a lack of lineage-defining sequence polymorphisms in this region, the exact breakpoint cannot be defined [19]). Considering that Delta_pp_ and BA.1_pp_ showed comparable sensitivity to antibody-mediated neutralization, one must postulate that the first half of the NTD does not contribute to the differential neutralization sensitivity of the Delta and Omicron BA.1 variants of SARS-CoV-2. 

In sum, our study reveals that the cell entry of XD and its inhibition by antibodies is very similar to that of BA.1. One would thus assume that BA.1 and XD target similar cells and tissues in infected patients and that XD, similar to BA.1, has reduced capacity to spread in the alveolar space and, potentially, to cause severe disease. To what extent this limitation might be rescued by the Delta variant-derived genetic “backbone” of XD remains to be examined within animal studies. However, the investigation of human patients infected with XD revealed evidence for mild disease [18], as expected for infection with Omicron BA.1. 

The high incidence of co-circulation of multiple SARS-CoV-2 lineages is a driver for recombination events that can result in recombinant SARS-CoV-2 lineages with unique virological characteristics. A key strategy to reduce the risk of recombination between SARS-CoV-2 lineages is the use of vaccines that establish broad immunity against multiple SARS-CoV-2 lineages. Most COVID-19 vaccines are based on SARS-CoV-2 lineages circulating in the early phase of the pandemic (such as B.1) [29]. However, in the past 2.5 years, several SARS-CoV-2 lineages emerged that acquired S protein mutations, which reduced the sensitivity to neutralization by antibodies elicited upon vaccination with these COVID-19 vaccines [2,9,12,30,31,32,33]. Multivalent vaccines, such as the recently approved bivalent vaccines by Moderna and BioNTech/Pfizer, which include the S proteins of SARS-CoV-2 B.1 lineage and the currently dominating BA.5 lineage as antigens, have been shown to increase neutralization breadth [34] and may thus reduce the likelihood of recombination events.

While the recombinant XD lineage of SARS-CoV-2 did not appear to have an advantage over BA.1 with respect to transmissibility and immune evasion and was thus not able to spread efficiently, future recombinant SARS-CoV-2 lineages may differ. As a matter of fact, another recombinant SARS-CoV-2 lineage, XBB, which is derived from the recombination of BJ.1 and BM.1.1 lineages (both constitute sublineages of the BA.2 lineage of the Omicron variant), appears to have some advantage over other currently circulating SARS-CoV-2 lineages, as it is presently increasing in incidence [35]. In this context, our study provides a blueprint for initial virological characterization and risk assessment of novel recombinant SARS-CoV-2 lineages.

Our study has the following limitations: Our results await confirmation with authentic SARS-CoV-2 XD, which was not available to us. Further, one could compare XD_pp_ and BA.1_pp_ neutralization by monoclonal antibodies used for COVID-19 therapy. However, no differences are to be expected since all clinically used monoclonal antibodies target the RBD.

## 4. Materials and Methods

### 4.1. Cell Culture

Vero cells stably expressing human TMPRSS2 (Vero-TMPRSS2) [36], 293T (human, female, kidney; ACC-635, DSMZ; RRID: CVCL 0063), Huh-7 (human, male, liver; JCRB Cat# JCRB0403; RRID: CVCL 0336, kindly provided by Thomas Pietschmann), and Vero cells (African green monkey kidney, female; CRL-1586, ATCC, kindly provided by Andrea Maisner) were cultured in Dulbecco’s modified Eagle medium (PAN-Biotech, Aidenbach, Germany), supplemented with 10% fetal bovine serum (Biochrom Berlin, Germany) and penicillin (100 U/mL)/streptomycin (0.1 mg/mL) solution (PAA Laboratories GmbH, Cölbe, Germany). Vero-TMPRSS2 further received 10 mg/mL blasticidin (Invivogen, San Diego, CA, USA). Calu-3 (human, male, lung; HTB-55, ATCC; RRID: CVCL 0609; kindly provided by Stephan Ludwig) and Caco-2 (human, male, colon; HTB-37, ATCC; RRID: CVCL 0025) cells were cultivated in the Minimum Essential Medium (Thermo Fisher Scientific, Waltham, MA, USA), supplemented with 10% fetal bovine serum (Biochrom), penicillin (100 U/mL)/streptomycin (0.1 mg/mL) solution (PAA Laboratories GmbH ), non-essential amino acid solution (PAA Laboratories GmbH), and 1 mM sodium pyruvate (Thermo Fisher Scientific). All cell lines were incubated in a humidified environment with 5% CO_2_ at 37 °C. Cell line validation was performed using STR-typing, amplification and sequencing of a cytochrome c oxidase gene fragment, microscopic analysis, and/or according to specific growth characteristics. In addition, all cell lines underwent routine testing for mycoplasma contamination. Transfection of 293T cells was carried out using calcium phosphate precipitation. 

### 4.2. Expression Plasmids

The plasmids pCAGGS-DsRed [36], pCAGGS-VSV-G (vesicular stomatitis virus glycoprotein) [37], pCG1-solACE2-Fc [38], pCG1- SARS-CoV-2 B.1 SΔ18 (codon-optimized, C-terminal truncation of 18 amino acid residues, GISAID Accession ID: EPI_ISL_425259) [2], and pCG1-SARS-CoV-2 BA.1 SΔ18 (GISAID Accession ID: EPI_ISL_6640919) [36] have been described previously. In order to generate the expression plasmids for SARS-CoV-2 XD SΔ18 (GISAID Accession ID: EPI ISL 12028907), five overlapping DNA strings were purchased (Thermo Fisher Scientific) and assembled by Gibson assembly. For this, gene strings, the BamHI/XbaI-digested pCG1 plasmid, and the GeneArtTM Gibson Assembly HiFi Master Mix (Thermo Fisher Scientific) were mixed and incubated according to the manufacturer’s instructions. The pCG1 plasmid was kindly provided by Roberto Cattaneo (Mayo Clinic College of Medicine, Rochester, MN, USA). A commercial sequencing service was used to verify all PCR-amplified sequences (Microsynth SeqLab, Göttingen, Germany). 

### 4.3. Production of Soluble ACE2

The preparation of soluble human ACE2 C-terminally fused to the Fc-portion of human immunoglobulin G (solACE2-Fc) has previously been described in detail [38]. Briefly, 293T cells were transfected with pCG1-solACE2-Fc. At 10h posttransfection, the medium was replaced, and cells were further incubated for 38 h. Then, the cell culture supernatant was collected and centrifuged to pellet cellular debris (2000× *g*, 10 min, 4 °C). Next, the clarified supernatant was concentrated (100×) using a Vivaspin protein concentrator column (molecular weight cut-off of 30 kDa; Sartorius, Göttingen, Germany), and concentrated solACE2-Fc was aliquoted and kept at −80 °C for further use.

### 4.4. ACE2 Binding

In order to determine the binding efficiency of the respective S proteins to ACE2, 293T cells were seeded in 6-well plates and transfected with the S protein-coding expression plasmid. Cells transfected with an empty plasmid served as control. At 24 h posttransfection, the culture medium was exchanged, and cells were further incubated. At 48 h posttransfection, the culture medium was removed, and cells were resuspended in PBS, transferred to 1.5 mL reaction tubes, and pelleted by centrifugation (600× *g*, 5 min, room temperature). After aspiration of the supernatant, cells were washed with PBS-B (PBS containing 1% bovine serum albumin) and pelleted by centrifugation. Again, the supernatant was removed, and cells were resuspended in PBS-B containing soluble solACE2-Fc (1:100). Samples were rotated for 1 h at 4 °C using a Rotospin test tube rotator disk (IKA, Staufen, Germany). Next, cells were pelleted, resuspended in PBS-B containing anti-human AlexaFlour-488-conjugated antibody (1:200; Thermo Fisher Scientific), and rotated for another 1h at 4 °C. Finally, cells were washed with PBS-B, fixed by incubation with 1% paraformaldehyde solution, washed again, and analyzed by flow cytometry using an LSR II flow cytometer (BD Biosciences, Franklin Lakes, NJ, USA). Data were further processed using the Flowing program (Turku Bioscience, Turku, Finland; https://bioscience.fi/services/cell-imaging/flowing-software/, accessed on 1 October 2022).

### 4.5. Production of VSV Pseudotypes 

Vesicular stomatitis virus (VSV) pseudotypes bearing SARS-CoV-2 S proteins were produced according to a published protocol [39]. At 24 h posttransfection, 293T cells transiently expressing the SARS-CoV-2 S protein, VSV-G (vesicular stomatitis virus glycoprotein, positive control), or no viral surface protein (negative control) were inoculated with VSV-G-transcomplemented VSVΔ*G(FLuc), which was kindly provided by Gert Zimmer (Institute of Virology and Immunology, Mittelhäusern, Switzerland) [40]. At 1 h postinoculation, the inoculum was removed, cells were washed with phosphate-buffered saline (PBS), and a culture medium supplemented with anti-VSV-G antibody (culture supernatant from I1-hybridoma cells; ATCC no. CRL-2700; 1:1000) was added (except for cells expressing VSV-G, which received the medium without the antibody). Cells were incubated for an additional 16-18h before culture supernatants were collected and cleared from cellular debris by centrifugation (4000× *g*, 10 min, room temperature). Clarified supernatants were aliquoted and kept at −80 °C until further use.

### 4.6. Immunoblot

To investigate S protein cleavage and particle incorporation, pseudotype particles bearing SARS-CoV-2 S proteins were loaded onto a sucrose cushion (20% *w*/*v* sucrose in PBS) and concentrated by high-speed centrifugation (13,300 rpm, 90 min, 4 °C). Following removal of the supernatant, pseudotype particles were lysed in 2× Sample buffer (0.03 M Tris-HCl, 10% glycerol, 2% SDS, 5% beta-mercaptoethanol, 0.2% bromophenol blue, 1 mM EDTA) and subjected to SDS-PAGE. Next, proteins were transferred onto nitrocellulose membranes (Hartenstein, Würzburg, Germany) using the Mini Trans-Blot Cell system (Bio-Rad, Hercules, CA, USA). Membranes were blocked by incubation in PBS-T (PBS containing 0.05% Tween-20) supplemented with 5% BSA for 30 min. After blocking, membranes were probed overnight at 4 °C with primary antibodies to S2 (1:2000 in PBS-T containing 5% BSA, rabbit, Biozol, Eching, Germany) or VSV-M (1:1000 in PBS-T containing 5% skim milk powder, mouse, Kerafast, Boston, MA, USA). The next day, membranes were washed three times with PBS-T for 10 min and probed for 1h at room temperature with horseradish peroxidase-conjugated secondary antibodies (both 1:2000 in PBS-T containing 5% skim milk powder, Dianova, Hamburg, Germany). Thereafter, membranes were washed three times with PBS-T. Membranes were developed using a homemade chemiluminescence solution (0.1 M Tris-HCl [pH 8.6], 250 g/mL luminol, 0.1 mg/mL para-hydroxycoumaric acid, and 0.3 percent hydrogen peroxide). For imaging, a ChemoCam imager equipped with the ChemoStar Professional software was used (Intas Science Imaging Instruments, Göttingen, Germany). The quantification of protein bands was performed using the ImageJ software (version 1.53C, National Institutes of Health, Bethesda, MD, USA; available at https://imagej.nih.gov/ij/, accessed on 1 October 2022) was utilized. For the analysis of S protein incorporation into VSV particles, total S protein signals (uncleaved, S0, and cleaved, S2) were normalized against their corresponding VSV-M signals. The resulting values were then compared using the B.1 S protein as a reference (set as 1). In order to quantify S protein cleavage, total S protein signals (uncleaved, S0, and cleaved, S2) were set to 100% for each S protein, and the relative amounts of S0 and S2 were calculated.

### 4.7. Transduction of Target Cells

At 24 h post-seeding (96-well format), target cells were inoculated with equal volumes of pseudotype particles and further incubated for 16–18 h. Transduction efficiency was assessed by quantification of virus-encoded firefly luciferase activity in cell lysates. For this, the cell culture supernatant was aspirated, and cells were lysed by incubation (30 min, room temperature) with PBS containing 0.5% Triton X-100 (Carl Roth, Karlsruhe, Germany). Subsequently, cell lysates were transferred into white 96-well plates, luciferase substrate (Beetle-Juice, PJK, Kleinblittersdorf, Germany) was added, and luminescence was recorded using a Hidex Sense plate luminometer (Hidex, Turku, Finland).

### 4.8. Neutralization Assay

Serum/plasma was collected at the Hannover Medical School (Medizinische Hochschule Hannover, MHH) and University Medicine Göttingen (UMG). The collection of samples was approved by the Institutional Review Board of MHH (8973_BO_K_2020) and the ethics committee of UMG (reference number: 8/9/20). Patient information is provided in Table S1. In total, four groups were analyzed: (i) Plasma of individuals vaccinated with two doses of the BNT162b2/Comirnaty vaccine without breakthrough infection (*n* = 9); (ii) plasma of individuals vaccinated with three doses of the BNT162b2/Comirnaty vaccine without breakthrough infection (*n* = 10); (iii) serum of individuals vaccinated with three doses of the BNT162b2/Comirnaty vaccine with breakthrough infection during the “Delta wave” in Germany (*n* = 7); (iv) plasma of individuals vaccinated with three doses of the BNT162b2/Comirnaty vaccine with breakthrough infection during the “Omicron wave” in Germany (*n* = 9). All serum/plasma samples were heat-inactivated at 56 °C for 30 min prior to their use, and neutralization assays were performed according to an established protocol [31,33]. 

Briefly, S-protein-containing pseudovirus particles were pre-incubated (30 min, 37 °C) with fourfold serial serum/plasma dilutions. Thereafter, mixtures were added to Vero cells (96-well format) and incubated for 16–18 h. Transduction efficiency was analyzed as described above. For data normalization, pseudovirus particles incubated with medium alone served as reference (=0% inhibition). Finally, the serum/plasma dilution that results in a half-maximal inhibition of transduction (neutralizing titer 50, NT50) was calculated using a non-linear regression model.

### 4.9. Cell–Cell Fusion Assay 

The quantification of S protein-induced cell–cell fusion was analyzed by a beta-galactosidase reconstitution assay [41]. At 24 h post-seeding (24-well format), 293T effector cells were cotransfected with expression plasmids for the SARS-CoV-2 S protein (or empty vector, control) and the beta-galactosidase alpha fragment, while 293T target cells were cotransfected with expression plasmids for ACE2 and beta-galactosidase omega fragment. Following an incubation period of 18 h, the culture medium was exchanged, and cells were further incubated for 6 h. Next, cells were washed and resuspended in a culture medium before effector cells (one well) and target cells (one well) were mixed and further incubated. After 24 h of co-culture, beta-galactosidase substrate (Gal-Screen, Thermo Fisher Scientific) was added, and cells were incubated for 90 min at room temperature before luminescence was recorded using a Hidex Sense plate luminometer (Hidex).

### 4.10. Protein Structures

For an illustration of the three-dimensional structure of the Delta-Omicron (XD)-S protein, the prefusion structure of monomeric SARS-CoV-2 S protein with one RBD in the so-called “up” conformation (PDB: 6VSB) [42] was colorized using UCSF ChimeraX (version 1.3rc202111192158, University of California, San Francisco, CA, USA; https://www.cgl.ucsf.edu/chimerax/, accessed on 1 October 2022).

### 4.11. Data Analysis

Data analysis was performed using Microsoft Excel (as part of the Microsoft Office software package, version 2019, Microsoft Corporation, Redmond, WA, USA) and GraphPad Prism 8 version 8.4.3 (GraphPad Software, San Diego, CA, USA). Statistical analysis was carried out by a two-tailed Student’s *t*-test (efficiency of ACE2 binding, cleavage efficiency, fusion assay, and cell entry mediated by S protein) or Kruskal–Wallis analysis with Dunns’ multiple comparison test (neutralization assay). Only p-values of 0.05 or less were considered statistically significant (*p* > 0.05, ns); *p* 0.05, *; *p* 0.01, **; *p* 0.001, ***).

## Data Availability

The data presented in this study are available on request from the corresponding authors.

## Supplementary Materials

The following supporting information can be downloaded at: https://www.mdpi.com/article/10.3390/ijms232214057/s1.
