# Supplementary material for: The SARS-CoV-2 Delta-Omicron Recombinant Lineage (XD) Exhibits Immune-Escape Properties Similar to the Omicron (BA.1) Variant
Source: Int J Mol Sci. 2022 Nov 14;23(22):14057. doi: 10.3390/ijms232214057 (PMC9696394; doi:10.3390/ijms232214057)
Supplement: Supplementary file 1 [file ijms-23-14057-s001.zip › ijms-2019914-supplementary.pdf]

Figure S1

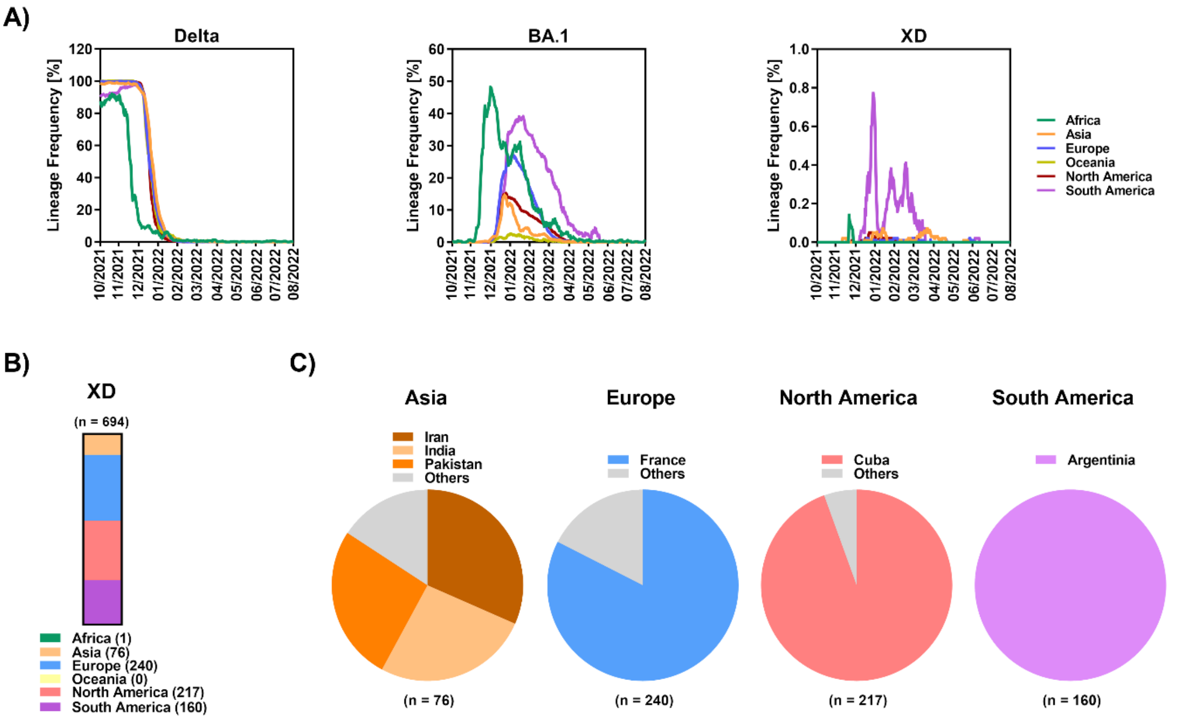

Figure S1. Epidemiology of SARS-CoV-2 Delta-Omicron (XD) recombinant. **(A)** Epidemiology of SARS-CoV-2 Delta variant (B.1.617.2 and sublineages), SARS-CoV-2 Omicron variant (BA.1 and sublineages), and SARS-CoV-2 Delta-Omicron recombinant (XD). **(B)** Global distribution of detected SARS-CoV-2 Delta-Omicron (XD) recombinant sequences. **(C)** Distribution of detected SARS-CoV-2 Delta-Omicron (XD) recombinant sequences per continent. All data were retreived from the CoV-Spectrum database (https://cov-spectrum.org).

Figure S2

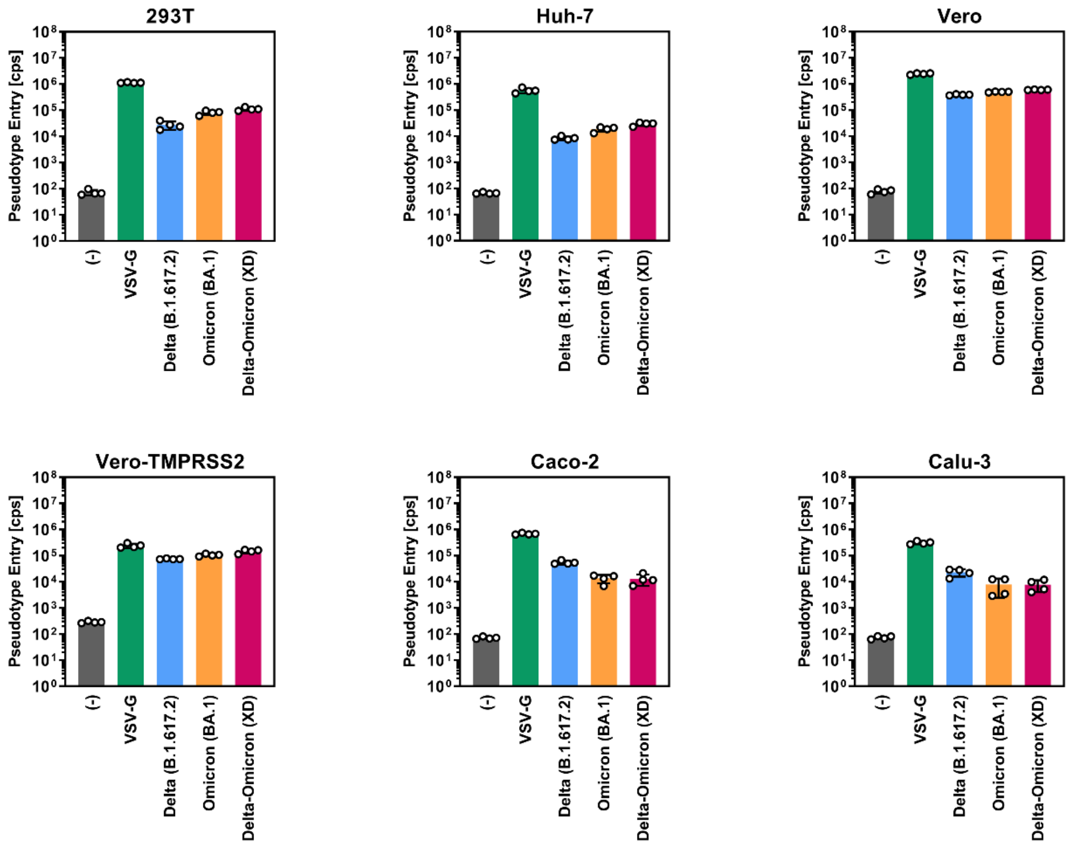

Figure S2. Host cell entry of SARS-CoV-2 Delta-Omicron (XD) recombinant. Representative unprocessed pseudotype entry data (shown as counts per second, cps) for particles bearing no viral surface protein (negative control), vesicular stomatitis virus glycoprotein (VSV-G, positive control) or the indicated SARS-CoV-2 S proteins. Mean data from single experiments conducted with four technical replicates are shown. Error bars indicate the standard deviation.

Figure S3

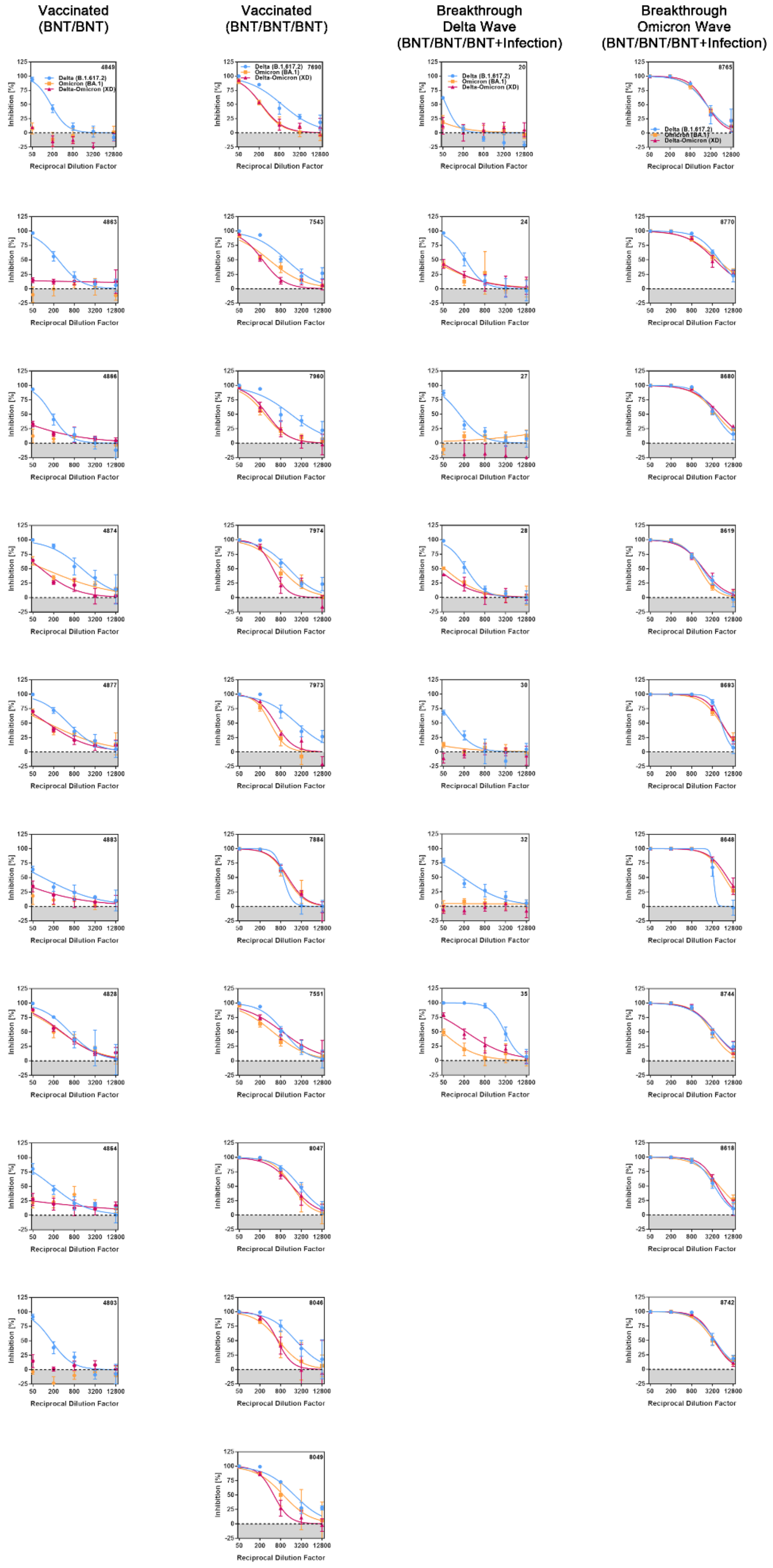

Figure S3. Immune evasion by SARS-CoV-2 Delta-Omicron (XD) recombinant. Individual neutralization data for sera/plasma from individuals that received two doses of the

Cormirnaty/BNT162b2 vaccine, three doses of the Cormirnaty/BNT162b2 vaccine, three doses of the Comirnaty/BNT162b2 vaccine and were infected during the "Delta wave" in Germany and three doses of the Comirnaty/BNT162b2 vaccine and were infected during the "Omicron wave" in Germany. For normalization, pseudoviruses entry in the absence of plasma was set as 0% inhibition (dashed line).

Table S1

Table S1. Information on sera/plasma.

ID	Gender	Age group (years)	Sample group	Vaccination	Time since last vaccination and sampling (days)	Documented SARS-CoV-2 infection? (yes/no, period, variant)	Time since first positive test and sampling (days)
4803	Female	35-44	Vaccinated	2x (BNT/BNT)	13	No[1], n.a., n.a.	n.a.
4828	Female	18-25		2x (BNT/BNT)	27	No[1], n.a., n.a.	n.a.
4849	Male	35-44		2x (BNT/BNT)	23	No[1], n.a., n.a.	n.a.
4863	Female	55-64		2x (BNT/BNT)	26	No[1], n.a., n.a.	n.a.
4864	Female	45-54		2x (BNT/BNT)	26	No[1], n.a., n.a.	n.a.
4866	Female	55-64		2x (BNT/BNT)	27	No[1], n.a., n.a.	n.a.
4874	Female	25-34		2x (BNT/BNT)	27	No[1], n.a., n.a.	n.a.
4877	Female	25-34		2x (BNT/BNT)	27	No[1], n.a., n.a.	n.a.
4883	Female	35-44		2x (BNT/BNT)	34	No[1], n.a., n.a.	n.a.
**7543**	Female	45-54		3x (BNT/BNT/BNT)	41	No[1], n.a., n.a.	n.a.
**7551**	Male	55-64		3x (BNT/BNT/BNT)	13	No[1], n.a., n.a.	n.a.
**7690**	Female	35-44		3x (BNT/BNT/BNT)	32	No[1], n.a., n.a.	n.a.
**7884**	Female	25-34		3x (BNT/BNT/BNT)	39	No[1], n.a., n.a.	n.a.
**7960**	Male	35-44		3x (BNT/BNT/BNT)	24	No[1], n.a., n.a.	n.a.
**7973**	Female	45-54		3x (BNT/BNT/BNT)	24	No[1], n.a., n.a.	n.a.
**7974**	Female	55-64		3x (BNT/BNT/BNT)	45	No[1], n.a., n.a.	n.a.

**8046**	Female	45-54		3x (BNT/BNT/BNT)	47	No[1], n.a., n.a.	n.a.
**8047**	Female	55-64		3x (BNT/BNT/BNT)	16	No[1], n.a., n.a.	n.a.
**8049**	Female	55-64		3x (BNT/BNT/BNT)	39	No[1], n.a., n.a.	n.a.
**UMG_20**	Female	75-84	**Vaccinated & Infected**	3x (no information)	unknown	Yes[2], Delta wave[3], unknown	13
**UMG_24**	Male	35-44		3x (no information)	85	Yes[2], Delta wave[3], unknown	3
**UMG_27**	Female	85-94		3x (no information)	unknown	Yes[2], Delta wave[3], unknown	15
**UMG_28**	Male	85-94		3x (no information)	>58	Yes[2], Delta wave[3], unknown	4
**UMG_30**	Female	75-84		3x (BNT/BNT/BNT)	63	Yes[2], Delta wave[3], unknown	11
**UMG_32**	Male	65-74		3x (no information)	unknown	Yes[2], Delta wave[3], unknown	12
**UMG_35**	Male	75-84		3x (no information)	unknown	Yes[2], Delta wave[3], unknown	11
**8618**	Female	25-34		Yes (BNT/BNT/BNT)	149	Yes[2], Early Omicron wave[4], unknown	25
**8619**	Male	25-34		Yes (BNT/BNT/BNT)	139	Yes[2], Early Omicron wave[4], unknown	23
**8648**	Male	35-44		Yes (BNT/BNT/BNT)	146	Yes[2], Early Omicron wave[4], unknown	22
**8680**	Female	35-44		Yes (BNT/BNT/BNT)	123	Yes[2], Early Omicron wave[4], unknown	52
**8693**	Female	45-54		Yes (BNT/BNT/BNT)	149	Yes[2], Early Omicron wave[4], unknown	48
**8742**	Female	45-54		Yes (BNT/BNT/BNT)	174	Yes[2], Early Omicron wave[4], unknown	19

**8744**	Male	25-34		Yes (BNT/BNT/BNT)	187	Yes[2], Early Omicron wave[4], unknown	32
**8765**	Female	35-44		Yes (BNT/BNT/BNT)	152	Yes[2], Early Omicron wave[4], unknown	22
**8770**	Female	35-44		Yes (BNT/BNT/BNT)	167	Yes[2], Early Omicron wave[4], unknown	26

[1]: Vaccinees were anti-Spike IgG negative before vaccination and tested negative for anti-NCP IgG after their last vaccination

[2]: SARS-CoV-2 infection was confirmed by real-time reverse transcriptase polymerase chain reaction

[3]: Germany, 10/2021 to 01/2022; samples were pre-screened for their specific neutralisation activity against $B.1_{pp}$, $B.1.617.2_{pp}$ and $BA.1_{pp}$.

[4]: Germany, 02/2022 to 05/2022; samples were pre-screened for their specific neutralisation activity against $B.1_{pp}$, $B.1.617.2_{pp}$ and $BA.1_{pp}$.

Abbreviations: ID, identifier; n.a., not applicable; BNT, BNT162b2/Comirnaty
